# Family Medicine Clerkship Teaching About Contraception: Meeting a Need

**DOI:** 10.7759/cureus.10394

**Published:** 2020-09-11

**Authors:** Dharam Persaud-Sharma, Joseph Burns, Kyle P Sellers, Marquita Samuels, Sarah Stumbar

**Affiliations:** 1 Internal Medicine, Kendall Regional Medical Center, Herbert Wertheim College of Medicine, Florida International University, Miami, USA; 2 Pediatrics, Cohen Children's Medical Center, New Hyde Park, USA; 3 Department of Translational Medicine, Herbert Wertheim College of Medicine, Florida International University, Miami, USA; 4 Department of Humanities, Health, and Society, Herbert Wertheim College of Medicine, Florida International University, Miami, USA; 5 Family Medicine, Herbert Wertheim College of Medicine, Florida International University, Miami, USA

**Keywords:** undergraduate medical education, women's health, family planning, contraception

## Abstract

Introduction: Women’s healthcare including preventive care, obstetric care, and family planning and counseling are important medical education topics that student physicians frequently encounter during their Family Medicine Clerkship in medical training; however, despite its brief emphasis, many students feel underprepared to counsel patients in a clinical setting. With this in mind, a case-based curriculum focused on women’s health was developed for students enrolled in the Family Medicine Clerkship.

Methods: During the 2018-2019 academic year, 127 students on the Family Medicine Clerkship at the Florida International University Herbert Wertheim College of Medicine (HWCOM) participated in three two-hour sessions on the topics of adolescent preventive health care, obstetric care, and contraceptive counseling. These sessions incorporated cases, role-play activities, and pre-session readings. An optional, anonymous, paper-based pre-session survey of Likert-type questions was administered prior to the first session and a post-session survey of Likert-type questions was given following the final session. IRB exemption was obtained.

Results: The pre-session survey showed that 41.67% of students agreed or strongly agreed that they were confident in their ability to perform female contraceptive counseling. After the sessions, 92.68% of students agreed or strongly agreed that the case addressing contraceptive counseling was of clinical value to their learning needs. Conclusion: Given that less than half of the students felt confident in their counseling abilities prior to these sessions, this curriculum is potentially addressing an unmet educational need. Students found this session to be relevant, indicating that a case-based discussion curriculum that incorporates both pre-session reading materials and role-play interactions may be a promising educational approach.

## Introduction

Between 2006 and 2010, nearly 43 million women between the ages of 15 and 44 reported receiving gynecologic care, including family planning services and Pap tests during the preceding year [1]. The role of the family physician in addressing women’s healthcare is well-established. For example, approximately one-third of pregnant women report seeking care from family physicians [2]. As two-thirds of women of reproductive age in the United States currently use some type of contraception, contraceptive counseling and management is an important skill for any future physician [3].

An analysis of 21 medical schools across the United States found that students looking to gain competency in contraceptive counseling often must undertake family planning electives [4]. Another series of four focus groups of medical students identified lack of time devoted to contraception in didactic education as a primary perceived barrier to education on this topic [5]. As patient-centered contraception counseling leads to increased use, gaining competency in this skill is important for students [6]. While the Society of Teachers of Family Medicine (STFM) National Clerkship Curriculum does not have specific objectives related to contraceptive counseling, this topic is generally addressed in preventive health visits, which are a focus of any Family Medicine curriculum [7].

Case-based discussion has been shown to be an effective teaching strategy in medical education [8]. A longitudinal case-based discussion curriculum that consisted of three two-hour sessions spanning multiple didactic days was implemented in order to ensure that students in the third year Family Medicine Clerkship at the Herbert Wertheim College of Medicine (HWCOM) at Florida International University in Miami were receiving adequate exposure to women’s healthcare. Topics in women's healthcare that were included in this curriculum included adolescent preventive health counseling, obstetric care, and contraceptive counseling. On the first day, students were introduced to a simulated patient to address her preventive health needs. The second session guided students through a simulated pregnancy cycle; outcomes from these sessions have been published [9]. The third session introduced students to contraceptive counseling as part of the simulated patients’ post-partum visit. Before participating in the session, students were provided with two articles for pre-reading materials [10,11]. The purpose of this study was to evaluate the enrolled students’ perceived need for and perception of this contraceptive counseling session.

## Materials and methods

During the 2018-2019 academic year, 127 students on the Family Medicine clerkship at the Florida International University HWCOM participated in three two-hour case-based learning sessions regarding adolescent preventive health care, obstetric care, and contraceptive counseling. Each session involved all 19-23 students completing their Family Medicine clerkship and was facilitated by a Family Medicine-trained faculty member with specific expertise in women’s health. The learning objectives for the three sessions are detailed in Table 1.

**Table 1:** Learning objectives for longitudinal contraception case-based learning sessions HEADSSS: Home, Education/Employment, Eating, Activities, Drugs, Sexuality, Suicide/Depression, and Safety

By the end of these sessions, students should be able to:
Day 1: Preventive Care
· Describe the HEADSSS exam and the essential elements of an adolescent preventative health visit.
· Understand the family physician’s role in well-patient care; and be able to discuss age- and sex-appropriate preventative care guideline.
Day 2: Obstetric Care
· Explain the management of first trimester bleeding, including the use of and indications for imaging, lab tests, medications, and surgical procedures.
· Articulate the pathophysiology and appropriate management of nausea/vomiting of pregnancy.
· Explain the difference between chronic hypertension in pregnancy and pre-eclampsia, including the diagnostic criteria of each.
· List the diagnostic criteria for gestational diabetes and its first-line treatment options, as well as potential complications that may impact the fetus.
· Articulate how obstetric care is in alignment with the principles of Family Medicine
Day 3: Post-Partum Care
· Counsel a young female patient regarding contraception options in the immediate post-partum period and list other post-partum preventative health concerns.

An optional, anonymous, paper-based pre-session survey of Likert-type questions evaluating students’ confidence regarding contraceptive counseling was administered prior to the first session, and a post-session survey of Likert-type questions assessing students’ perceptions of the session was given following the final session. Data was categorized based on whether or not students had already completed their obstetrics/gynecology (OBGYN) clerkship. IRB exemption was obtained.

## Results

All 127 students in the clerkship participated in the case-based learning sessions; 96.3% completed the pre-session survey, and 59.8% completed the post-session survey. At the time of participation, 61 students had not completed their OBGYN clerkship and 54 had. Among the students who had not already completed their OBGYN clerkship, 36.01% agreed or strongly agreed that they were confident in their ability to perform male contraceptive counseling, and 41.67% felt similarly about their ability to perform female contraceptive counseling.

Among the students who had completed their OBGYN clerkship, 50.0% agreed or strongly agreed that they were confident in their ability to perform male contraceptive counseling, and 74.1% felt similarly about their ability to perform female contraceptive counseling. Responses to the pre-session questions are found in Table 2.

**Table 2:** Students’ responses to pre-session survey, categorized based on whether or not students had already completed their obstetrics/gynecology (OBGYN) clerkship ^a^n=61, response rate: 100% ^b^n=52, response rate: 96.3% ^c^n=60, response rate: 98.4% ^d^n=54, response rate: 100%

Statement	Number (%)
I am confident in my ability to perform male contraceptive counseling.	
Students who had not already completed the OBGYN clerkship^a^	
Strongly agree	3 (4.91)
Agree	19 (31.1)
Neutral	25 (41.0)
Disagree	14 (23.0)
Strongly Disagree	0 (0.00)
Students who had already completed OBGYN clerkship^b^	
Strongly Agree	5 (9.62)
Agree	21 (40.4)
Neutral	16 (30.8)
Disagree	9 (17.3)
Strongly Disagree	1 (1.92)
I am confident in my ability to perform female contraceptive counseling.	
Students who had not already completed their OBGYN clerkship^c^	
Strongly agree	1 (1.67)
Agree	24 (40.0)
Neutral	23 (38.3)
Disagree	12 (20.0)
Strongly Disagree	0 (0.00)
Students who had already completed their OBGYN clerkship^d^	
Strongly Agree	13 (24.1)
Agree	27 (50.0)
Neutral	12 (22.2)
Disagree	2 (3.70)
Strongly Disagree	0 (0.00)

93.6% of students agreed or strongly agreed that the pre-session readings provided them with clinically relevant knowledge, and 96.1% of students agreed or strongly agreed that the in-person sessions helped them apply the information from the pre-session readings. 92.68% of the students agreed or strongly agreed that the case was of clinical value to them and their learning needs. Responses to the post-session questions can be found in Table 3.

**Table 3:** Students’ responses to post-session questions ^e^n=78, response rate: 61.5% ^f^n=76, response rate: 59.8% ^g^n=82, response rate: 64.6%

Statement	Number (%)
The pre-session readings provided me with clinically relevant knowledge^e^	
Strongly Agree	34 (43.6)
Agree	39 (50.0)
Neutral	5 (6.41)
Disagree	0 (0.00)
Strongly Disagree	0 (0.00)
The Natalie case study helped me to apply the information from the pre-session readings^f^	
Strongly Agree	35 (46.1)
Agree	38 (50.0)
Neutral	3 (3.95)
Disagree	0 (0.00)
Strongly Disagree	0 (0.00)
Natalie's case was of clinical value to me and my learning needs^g^	
Strongly Agree	38 (46.34)
Agree	38 (46.34)
Neutral	6 (7.32)
Disagree	0 (0.00)
Strongly Disagree	0 (0.00)

## Discussion

The results of the pre-session survey revealed that many students on the Family Medicine Clerkship were not confident in their ability to perform contraceptive counseling. Although students who had already completed the OBGYN clerkship were more confident than those who had not, approximately half of these students still did not feel confident in their ability to provide male contraceptive counseling. This suggests that there is room to improve the current curriculum, particularly regarding male contraceptive counseling; male contraceptive counseling was not a focus of our curriculum.

Additionally, even after the OBGYN clerkship, approximately one quarter of students reported that they did not feel confident in their ability to provide female contraceptive counseling; this suggests that the OBGYN clerkship alone is not sufficient in providing medical students with this education.

Students had an overwhelmingly positive reaction to this curriculum, with over 90% of students agreeing that the sessions were of value to their learning needs. This positive reaction may indicate that education regarding contraception is generally insufficient within medical school curricula, especially during clinical years when students are tasked with independently counseling patients. The fact that students found this session to be relevant indicates that a case-based discussion curriculum that incorporates both pre-session reading materials and role-play interactions may be a promising tool in the education of medical students on family planning and counseling.

These results support findings from the literature which indicate that medical students find that the case-based learning model enhances their learning [12,13]. There is not much literature regarding the effectiveness of case-based learning specifically regarding contraceptive counseling and family planning; however, one study by Weiss et al. demonstrated that case-based learning is useful in education regarding women's health in general [14]. Research has not been conducted on case-based curricula that incorporate role-play interactions and pre-session reading.

Limitations of this study include its cross-sectional design, which precludes an analysis of the long-term impact of this curriculum. Self-selection of the survey may have introduced bias into data to complete the post-session survey. Also, the questions from the pre-session survey were not included in the post-session survey. As such, statistical analysis could not be completed to compare pre-session and post-session confidences. 

## Conclusions

This study provides insight into a unique educational approach to contraception counseling and family planning that can be easily implemented in medical school education. The approach was novel in that it included both student-developed pre-session reading materials and role-play simulations during the educational encounter to improve student comfort with counseling patients in a clinical environment. The results of this study indicated that this multi-dimensional approach may be useful in teaching medical students about women’s healthcare needs from contraception, hygiene, and throughout pregnancy. Further utilization of this approach will allow students to develop the skills necessary to successfully counsel their patients. Future research should look more directly at student confidence by including related questions in the post-session survey. Further adaptations of this curriculum would be the incorporation of a similar clinical encounter as a structured, end of the year observed clinical skills examination (OSCE).
